# Draft genome sequences of *Escherichia coli* isolated from fecal samples of migratory birds from zoonotic wetland sources in Bangladesh

**DOI:** 10.1128/mra.00326-26

**Published:** 2026-06-04

**Authors:** Most. Nahida Khatun, Sourav Chakraborty, Md. Iftehimul, Md. Alimul Islam, Rokshana Parvin, Muhammad Tofazzal Hossain, Emdadul Haque Chowdhury

**Affiliations:** 1 Department of Pathology, Faculty of Veterinary Science, Bangladesh Agricultural University 54492, Mymensingh, Bangladesh; 2 Department of Microbiology and Hygiene, Faculty of Veterinary Science, Bangladesh Agricultural University 54492, Mymensingh, Bangladesh; 3 Institute of Biotechnology, Bangladesh Agricultural University 54492, Mymensingh, Bangladesh; University of Maryland Baltimore, Baltimore, Maryland, USA

**Keywords:** *Escherichia coli*, migratory birds, WGS, Bangladesh

## Abstract

We report the draft genome sequences of four *Escherichia coli* isolates obtained from migratory birds in Bangladesh. Genome sizes ranged from 4,772,145 to 4,873,191 bp, with 72–81× coverage and approximately 51% GC content, providing enhanced understanding of *E. coli* diversity in wildlife reservoirs.

## ANNOUNCEMENT

*Escherichia coli* is widely used to study the evolution and spread of antimicrobial resistance (AMR) (1, 2), a growing global health threat, particularly in low- and middle-income countries such as Bangladesh (3). Beyond clinical and agricultural settings, migratory birds play a crucial ecological role in the dissemination of resistant bacteria (4). Due to their long-distance movements across continents and diverse habitats, migratory birds can act as reservoirs and carriers of AMR-associated *E. coli*, facilitating environmental persistence and transboundary spread of resistance (5).

The fecal samples of migratory birds were collected from Tanguar (25.15° N, 91.07° E) and Hakaluki Haor (24.67° N, 92.05° E) in Bangladesh. The freshly voided 1 g fecal samples were enriched in nutrient broth (NB; HiMedia, India) and incubated overnight at 37°C, then streaked onto eosin methylene blue (Oxoid, UK) agar and incubated again at 37°C. Colonies exhibiting a green metallic sheen were presumed to be *E. coli* and were subcultured for purity. An isolated colony was grown overnight in NB at 37°C, and genomic DNA was isolated using the QIAamp DNA Mini Kit (QIAGEN, Hilden, Germany) following the manufacturer’s protocol. DNA concentration and quantity were determined using NanoDrop (Thermo Fisher Scientific, USA) (6). The Illumina DNA Prep Library Preparation Kit (Illumina, Inc., San Diego, CA, USA) was used to create sequencing libraries, and sequencing was performed on an Illumina NovaSeq 6000. FastQC v0.12.1 (7) was used to verify the quality of sequence reads, and Fastp v0.24.0 (8) was used to filter them. *De novo* assembly was performed using SPAdes v4.0.0 (9). The resultant post-quality-filtered assemblies were assessed with QUAST v5.3.0 (10), and ANI values were estimated using the OrthoANI algorithm implemented in EZBioCloud (11) as a reference (accession number GCF_000005845.2). Genomes were annotated using the NCBI Prokaryotic Genome Annotation Pipeline v6.9 (12). Unless otherwise noted, all analyses were performed with the default settings.

The assembled genomes of *E. coli* strains contain 4,772,145–4,873,191 bp, spanning contigs 182, 188, and 197, with sequencing depths of 81×, 77×, and 72×, respectively. The GC content was approximately 51% for all strains. Assembly statistics revealed L50 values of 10, 12, and 13, and N50 values of 162.8, 157.7, and 139.7 kb. Detailed genomic characteristics are summarized in Table 1.

**TABLE 1:** Information about the genomic features of *E. coli* strains obtained from the feces of migratory birds in Bangladesh

	Tanguar haor	Hakaluki haor	
Parameters	*E. coli* MBD03	*E. coli* MBD04	*E. coli* MBD05
BioSample accession number	SAMN54195609	SAMN54195610	SAMN54195611
GenBank accession number	GCA_055330045.1	GCA_055329985.1	GCA_055329945.1
SRA accession number	SRS27513104	SRS27513101	SRS27513103
Nucleotide accession number	JBUZWA000000000	JBUZVZ000000000	JBUZVY000000000
Number of reads generated (million)	1.4	1.3	1.2
Contigs	182	188	197
Genome length (bp)	4,873,191	4,772,145	4,773,607
GC content (%)	51	50.5	50.5
Genes (total)	4,821	4,722	4,731
CDSs (total)	4,745	4,640	4,645
Genes (coding)	4,635	4,498	4,504
CDSs (with protein)	4,635	4,498	4,504
Genes (RNA)	76	82	86
rRNAs	2, 1, 1 (5S, 16S, 23S)	3, 4, 1 (5S, 16S, 23S)	4, 5, 1 (5S, 16S, 23S)
Complete rRNAs	1, 1 (16S, 23S)	1, 1, 1 (5S, 16S, 23S)	1, 1 (16S, 23S)
Partial rRNAs	2 (5S)	2, 3 (5S, 16S)	4, 4 (5S, 16S)
tRNAs	61	64	66
ncRNAs	11	10	10
Pseudogenes (total)	110	142	141
CDSs (without protein)	110	142	141
Pseudogenes (ambiguous residues)	0 of 110	0 of 142	0 of 141
Pseudogenes (frameshifted)	35 of 110	51 of 142	49 of 141
Pseudogenes (incomplete)	67 of 110	85 of 142	85 of 141
Pseudogenes (internal stop)	22 of 110	32 of 142	32 of 141
Pseudogenes (multiple problems)	12 of 110	23 of 142	22 of 141
CRISPR arrays	5	4	4
Average nucleotide identity (%)	99.3	99.31	99.31

## Data Availability

All raw *Escherichia coli* genome sequencing data were uploaded to NCBI, GenBank, and SRA under BioProject number PRJNA1390686.
